# Quantifying Argonaute 2 (Ago2) expression to stratify breast cancer

**DOI:** 10.1186/s12885-019-5884-x

**Published:** 2019-07-19

**Authors:** M.C. Casey, A. Prakash, E. Holian, A. McGuire, O. Kalinina, A. Shalaby, C. Curran, M. Webber, G. Callagy, E. Bourke, M. J. Kerin, J. A. Brown

**Affiliations:** 1 Discipline of Surgery, School of Medicine, Lambe institute for Translational Research, National University of Ireland, Galway, Ireland; 2 Discipline of Pathology, School of Medicine, Lambe Institute for Translational Research, National University of Ireland, Galway, Ireland; 3 School of Mathematics, Statistics and Applied Mathematics, National University of Ireland, Galway, Ireland

**Keywords:** Ago2, EIF2C2, Staining, Tumour, IHC, Pattern, Argonaute 2, miRNA, microRNA, Breast, Cancer, Midbody, Cytoplasmic, Intensity, Expression, mRNA, Biomarker, Survival, Disease, Relapse, Disease, DFS, RFS, Gene, Amplification

## Abstract

**Background:**

Argonaute-2 (Ago2) is an essential component of microRNA biogenesis implicated in tumourigenesis. However Ago2 expression and localisation in breast cancer remains undetermined. The aim was to define Ago2 expression (mRNA and protein) and localisation in breast cancer, and investigate associations with clinicopathological details.

**Methods:**

Ago2 protein was stained in breast cancer cell lines and tissue microarrays (TMAs), with intensity and localization assessed. Staining intensity was correlated with clinicopathological details. Using independent databases, Ago2 mRNA expression and gene alterations in breast cancer were investigated.

**Results:**

In the breast cancer TMAs, 4 distinct staining intensities were observed (Negative, Weak, Moderate, Strong), with 64.2% of samples stained weak or negatively for Ago2 protein. An association was found between strong Ago2 staining and, the Her2 positive or basal subtypes, and between Ago2 intensity and receptor status (Estrogen or Progesterone). In tumours Ago2 mRNA expression correlated with reduced relapse free survival. Conversely, Ago2 mRNA was expressed significantly lower in SK-BR-3 (HER2 positive) and BT-20 (Basal/Triple negative) cell lines. Interestingly, high levels of Ago2 gene amplification (10–27%) were observed in breast cancer across multiple patient datasets. Importantly, knowledge of Ago2 expression improves predictions of breast cancer subtype by 20%, ER status by 15.7% and PR status by 17.5%.

**Conclusions:**

Quantification of Ago2 improves the stratification of breast cancer and suggests a differential role for Ago2 in breast cancer subtypes, based on levels and cellular localisation. Further investigation of the mechanisms affecting Ago2 dysregulation will reveal insights into the molecular differences underpinning breast cancer subtypes.

**Electronic supplementary material:**

The online version of this article (10.1186/s12885-019-5884-x) contains supplementary material, which is available to authorized users.

## Background

MicroRNA (miRNA) are 18–22 nucleotide long non-coding RNA molecules regulating gene expression and function [1–3]. miRNA have a known role in the progression and regulation of many cancers, and have demonstrated utility as cancer biomarkers [4–8]. Argonaute-2 (Ago2) is essential for RNA interference (RNAi) and miRNA biogenesis and function [9–12]. One of four mammalian Ago proteins, Ago2 is the only member of the family to play an essential role as a core protein in the RNA-induced silencing complex (RISC) [13–15]. Within this complex, Ago2 directly binds small RNA molecules, with its slicer activity producing mRNA cleavage or translational repression of the complementary mRNA, causing gene silencing [16–19]. Recently it has been noted that dysregulation of the miRNA processing machinery components can also play a crucial role in cancer [20–24], and may serve as prognostic markers [9, 25–27].

To date, alteration in the Ago2 gene expression (both up and down-regulation) has been identified across multiple cancer types, including colorectal, urothelial, prostate and melanoma [16, 23, 28–32]. In breast cancer, single-nucleotide polymorphisms of Ago2 have been associated with changes in disease free survival (DFS) and overall survival (OS) [16, 33, 34]. Interestingly, Ago2 expression has been shown to be regulated by the Estrogen receptor (ER) mediated pathway, suggesting a Ago2 may have a different role in role in the ER positive subtypes (Luminal A and Luminal B) [20, 29]. In addition to the characterised role of Ago2 in miRNA biogenesis, it was recently reported that Ago2 interacts directly with Tip60 playing an important role in the Dicer- and Drosha-dependent DNA double-strand break (DSB)–induced small RNAs (diRNA) mediated response to DNA damage, further expanding its cellular roles [1–3]. Furthermore, the miRNA independent role of Ago2 in regulating the transcription of the tumour metastasis factor focal adhesion kinase (FAK), suggests another role for Ago2 in mediating tumour progression [4–8]. Together, these results indicate that Ago2 could play a role in cancer progression or response to treatments. While Ago2 plays an essential role in the regulation of molecules and pathways with known involvements in the initiation and progression of breast cancer, Ago2 levels in breast cancer have not been investigated or correlated with key clinicopathological criteria (such as survival).

The aim of this work was to investigate and quantify Ago2 dysregulation (mRNA and protein) in breast cancer. After measuring and quantifying the levels of Ago2 in a Tissue Microarray (TMA), the results were correlated with key clinicopathological criteria (including age, stage, subtype, tumour size and survival outcomes). An independent breast cancer gene expression database was then used to investigate if the correlations observed in the TMA matched changes observed in Ago2 gene expression.

## Methods

### Cell culture conditions

Human breast cancer cell lines (MCF10A, T47D, BT-474, SK-BR3, BT-20) were cultured at 37 °C and 5% CO2, in media as directed by the American Type Culture Collection repository (ATCC). All cell lines were originally purchased from ATCC.

### Immunoblotting

Whole cell lysates were prepared using RIPA buffer (Sigma) containing a protease inhibitor cocktail (Roche). Whole cell lysates were re-suspended in reducing sample buffer, boiled and separated by SDS-PAGE. Proteins were transferred to nitrocellulose membranes (Life Technologies) by standard procedures. Indicated proteins detected using anti-Ago2 antibody (ab57113, Abcam) and anti-Actin by (A2066-Sigma).

### Immunofluorescence microscopy

Cells were prepared and stained as using standard techniques. Briefly, cells were fixed in 4% paraformaldehyde followed by permeabilisation in 0.05% Triton-X-100 and then blocked in PBS/1% BSA. Cells were incubated with anti-Ago2 (ab57113, Abcam) for 2 h at 37 °C, washed and incubated with Alexa Fluor® 594-conjugated anti-mouse secondary antibody and counterstained with 1R,2R- diaminocyclohexane(trans-diacetato) (dichloro) platinum(IV) (DAPI) (blue). Images were acquired with using an Olympus BX61Research System Microscope, with F-View® Imaging System. Olympus CellSens™ Dimension software was used for digital image capture and analysis. Z-stack images (10–15 layers) 0.2um apart were captured and merged using Maximum intensity projection. All images were captured using the same sensitivity and exposure times across cell lines.

### Immunohistochemical staining

Cell lines for immunohistochemical staining were fixed using − 20 °C Methanol. Cells were blocked in 2.5% Normal Horse Serum and probed using Ago2 (ab57113, Abcam) primary antibody. Cells were then stained using the Anti-Mouse Ig ImmPRESS™ Excel Staining Kit (Vector Laboratories) used as per manufacturers instructions. Haematoxylin was used as a counterstain and cells fixed in DPX (Sigma-Aldrich). The TMA was stained for Ago2 using the ImmPACT/ImmPRESS DAB Immunohistochemistry as per manufacturers instructions.

### Tissue microarray (TMA)

Clinical breast tissue samples comprised core biopsies, wide local excisions and mastectomy specimens received by the Galway University Hospital pathology department (1999–2005) which were used to construct a consecutive tissue microarray, based on breast cancer diagnosis and availability of biopsy tissue in the paraffin block. A core (0.6 mm diameter) of formalin-fixed paraffin-embedded tissue was used to construct the TMA, as previously described [9–12]. Tumour cells in each section were confirmed by haematoxylin and eosin staining by a clinical pathologist. Pathological data was collected from the clinical pathology reports. Images of the Ago2 stained sections were captured using an Olympus VS120 Digital Scanner with a 40x objective and images processed using OlyVIA software (v2.8).

### TMA patient cohort

This study group consists of consecutively collected breast cancer patients treated at a tertiary referral unit (Galway University Hospital) entered into a prospectively maintained database (1999–2005). Only patients with a definitive subtype were included. Multiple clinical-pathological details were selected as indicated and used for further analysis. Tumours were staged according to the International Union against Cancer’s Tumour-Node-Metastasis (TNM) classification and histologically subtyped according to WHO guidelines. A total of 328 patients had Ago2 staining results with matched complete clinical information, including survival and outcome data for 327 patients. Table 1 describes the collected clinicopathological characteristics of the cohort.

**Table 1:** Breast Cancer Tissue Microarray: Clinicopathological Details

	Stained for Ago2 N (%)
Age	
Pre/Peri-menopausal	85 (29.2%)
Post-menopausal	206 (70.8%)
Total (n=)	291
Subtype	
Luminal A	119 (36.3%)
Luminal B	29 (8.8%)
Her2 positive	18 (5.5%)
Basal	60 (18.3%)
Unknown	102 (31.1%)
Total (n=)	328
Receptor Status	
ER Positive	197 (64.5%)
ER Negative	108 (35.4%)
Total (n=)	305
PR positive	182 (60.3%)
PR Negative	120 (39.7)
Total (n=)	302
Her2 positive	46 (20.3%)
Her2 negative	181 (79.7%)
Total (n=)	227
Stage	
1	104 (33.1%)
2	156 (49.7%)
3	26 (8.3%)
4	28 (8.9%)
Total (n=)	314
N Score	
0	143 (48%)
1	85 (28.5%)
2	45 (15.1%)
3	25 (8.4%)
Total (n=)	298
Metastatic disease	
No	289 (95.4%)
Yes	14 (4.6%)
Total (n=)	303

### Subtype definitions

Breast cancer molecular subtypes were defined based using standard accepted markers: Luminal A [ER and/or PR positive, HER2 negative, Ki-67 low (< 20%)]; Luminal B [ER and/or PR positive, HER2 positive or [ER and/or PR positive, HER2 negative, Ki-67 high (> 20%)]; HER2-overexpressing (ER and PR negative, HER2 positive); Triple negative (ER, PR and HER2 negative). The HER2 receptor status was identified by immunohistochemistry with any inconclusive results confirmed using a FISH test.

### TMA scoring

**Fig. 1:**
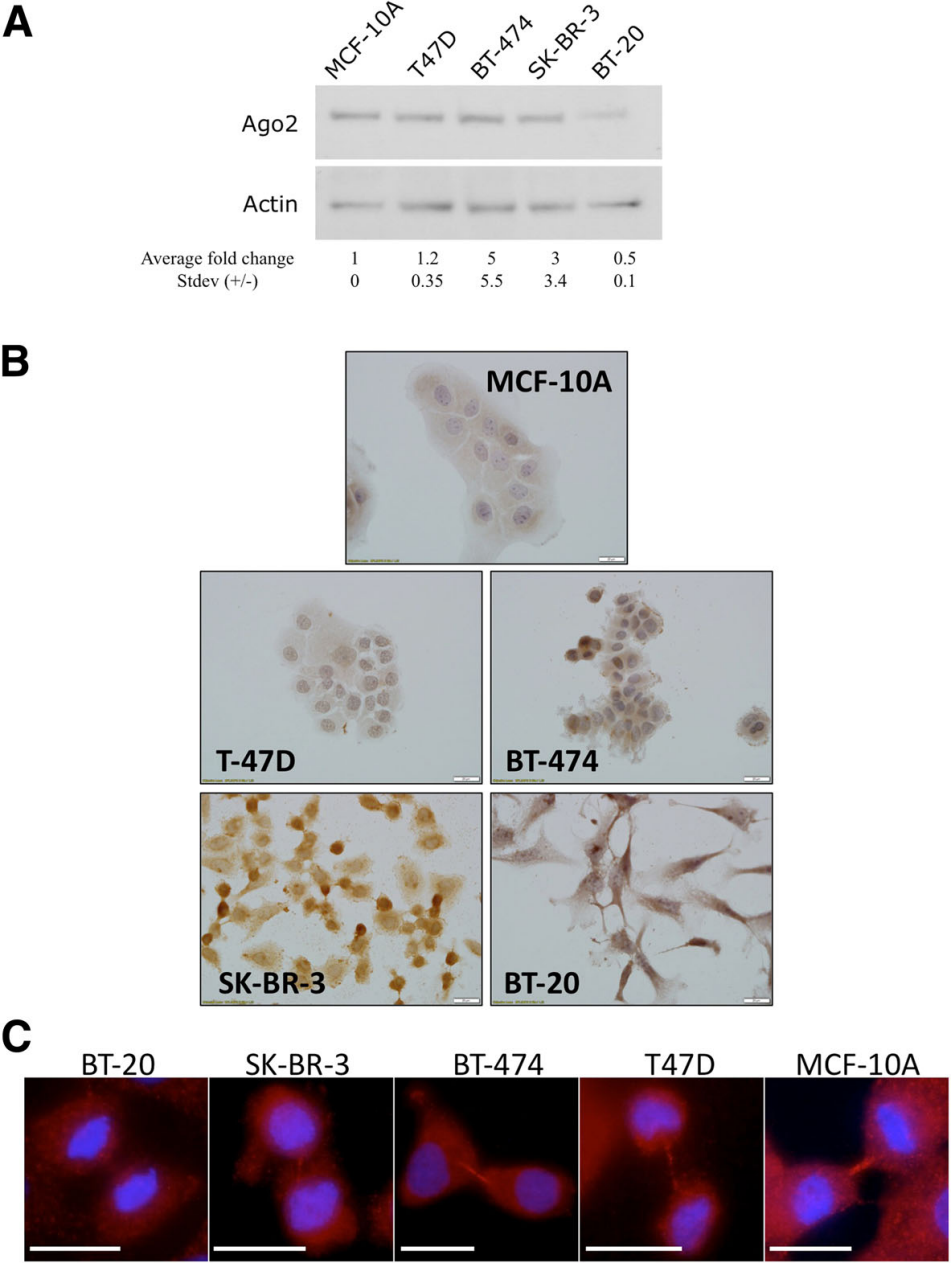
Ago2 protein expression in breast cancer cell lines. **a** Immunoblot of Ago2 protein levels in indicated breast cancer cell lines. Actin loading control. Numbers indicate actin normalised values (fold difference compared to MCF10A) of Ago2 protein. *n* = 2. **b**. Representative Ago2 IHC staining in breast cancer cell lines. Representative images from multiple experiments (*n* = 3). **c**. Breast cell lines are stained for Ago2 (red) and DAPI (blue). Representative midbody staining for Ago2 (dividing cells only) highlighted (right panels). Scale bar, 20um. Representative images from multiple experiments (*n* = 3)

The scoring system developed (in collaboration with clinical pathologists) was based on the observed pattern of Ago2 cytoplasmic staining (in control and breast cancer specimens), with staining intensities placed in categorises (Negative, Weak, Moderate or Strong) (See Fig. 1a and Additional file 1: Figure S1). TMA images (Ago2 stained) were scored in an independent blinded analysis (by two independent researchers, with further validation performed by a trained practicing clinical pathologist). Independent analysis of the TMA scoring was performed by the study biostatisticians.

### Real-time quantitative polymerase chain reaction (RQ-PCR)

TaqMan assays were used as per manufacturers instructions for the relative quantification PCR (RQ-PCR) of our target gene Ago2 (Hs01085579_m1), and the endogenous control genes used were Mitochondrial Ribosomal Protein L19 (MRPL19) (Hs00608519_m1) and Peptidylprolyl Isomerase A (PPIA) (Hs99999904_m1) [13–15]. Assays were performed using an AB7900HT (Applied Biosystems), using standard conditions as per the manufactures instructions. mRNA expression levels were normalized using endogenous controls PPIA and MRPL-19. Raw fluorescence data from RQ-PCR was exported into the software package qBase plus and relative quantification performed.

### Statistical analysis

Gene expression values obtained from cell line assays of mRNA expression (RQ-PCR) were exported from qBase to Minitab (v17) statistical software for analysis. As the mRNA expression data was not normally distributed, all results were log transformed for analysis. One-way ANOVA was used to test for significance between cell line mRNA expression values. Significance was reached if *p* ≤ 0.05.

The generated data (Ago2 TMA staining, clinicopathological and survival data) were statistically analysed, associations between categorical variables were evaluated by applying Cochran-Mantel-Haenszel tests (as appropriate), measured via Goodman and Kruskal’s Lambda statistic and Cramer’s V statistic. ANOVA or non-parametric alternative Kruskal-Wallis H tests were applied to test for differences in continuous variables by categories of staining. S series of Mann-Whitney tests were used with Bonferroni correction to control Type I error rate to calculate the effect size of significant differences. Significance was reached if p ≤ 0.05. To assess the relationship between Ago2 staining and the indicated clinicopathological variables for Overall Survival response and Disease Free Survival response Complete-Case Cox PH regression models were used. The R statistical program, and packages, were used for analysis.

### Kaplan-Meier plotter analysis

As previously described [35], the prognostic value of Ago2 mRNA expression was investigated using Breast Cancer database of The Kaplan-Meier Plotter (incorporating gene expression and clinical data) (http://kmplot.com/analysis/) [16–19]. At use, data on 5143 breast cancer patients was available (December 2017). Specific parameters used as previously described [35]: Gene: EIF2C2 (Search: AGO2) (Affy ID: 225827_at); Split patients by: median; Survival: Disease Free Survival (DFS *n* = 1402) or Overall Survival (OS, *n* = 626); Follow up threshold: all; Censure at threshold, selected; Use only JetSet best probe set, selected; Quality control, Remove redundant samples: checked; Array quality control: exclude biased arrays selected; Intrinsic subtype, selected as indicated.

### cBioPortal analysis

As previously described [35], somatic mutations and copy number changes to the Ago2 gene were investigated using the cBioPortal database [The Cancer Genome Atlas, Breast Cancer dataset] (http://www.cbioportal.org/) [20–24]. Specific Search parameters (as previously described [35]): Select Studies: Breast Cancer; Studies Select all: As of December 2017 9 studies available [Adenoid Cystic Carcinoma of the Breast (MSKCC, J Pathol. 2015; 12 samples), Breast Cancer (METABRIC, Nature 2012 & Nat Commun 2016; 2509 samples), Breast Invasive Carcinoma (British Columbia, Nature 2012; 65 samples), Breast Invasive Carcinoma (Broad, Nature 2012; 103 samples), Breast Invasive Carcinoma (Sanger, Nature 2012; 100 samples), Breast Invasive Carcinoma (TCGA, Provisional; 1105 samples), Breast cancer patient xenografts (British Columbia, Nature 2014; 29 samples), Mutational profiles of metastatic breast cancer (France, 2016; 216 samples), The Metastatic Breast Cancer Project (Provisional, October 2017; 103 samples)]; Select Data Type Priority: Mutation and CAN selected; Enter Gene Set: Ago2. Results returned following query submission were: copy number variation (CNV) and genomic alterations (including somatic mutations).

### Ethical approval

Ethical approval granted by University College Hospital Galway and National University of Ireland Galway (approvals C.A.151, C.A.1012). All patients included in the study had breast cancer confirmed histologically. A prospectively maintained breast cancer database provided the clinicopathological data. The Galway University Hospitals Clinical Research Ethics Committee approved use of patient material (2008 meeting; approval C.A.151; 2014 approval C.A.1012).

### Data availability

The produced datasets are available, from the corresponding author, on reasonable request.

## Results

### Ago2 expression and localisation in breast cancer cell lines

To investigate Ago2 expression in breast cancer, cell lines representing the four common molecular subtypes: T47D (Luminal A), BT-474 (Luminal B), SK-BR-3 (HER2 positive), BT-20 (Basal/Triple negative), and one non-cancer non-tumourigenic control breast epithelial cell line (MCF-10A) were profiled. The cell lines were assayed for Ago2 mRNA expression (Additional file 1: Figure S1A). T47D (Luminal A) was found to express Ago2 mRNA at significantly higher levels than either the SK-BR-3 (HER2 positive) or BT-20 (Triple Negative) cell lines (Anova *p* = 0.005). To further characterize Ago2 expression, the levels of Ago2 protein in a panel of subtype representative breast cancer cell lines was profiled (Fig. 1a). In agreement with the mRNA levels, the BT-20 line displayed distinctly lower total Ago2 protein expression than the other cell lines (MCF-10A, T47D, BT-474, SK-BR-3). Interestingly, only the SKBR-3 cell line displayed disagreement between the low mRNA and high protein expression levels indicating the presence of a highly stable Ago2 protein in these cells. In addition to profiling the total levels of Ago2, localisation of Ago2 protein in the breast cancer cell lines was explored. According to the Human Protein Atlas Ago2 localises predominantly to the nucleus and cell-cell junctions in 3 distinct cancer types, with breast cancer localisation unknown [9, 25–27]. Investigating Ago2 protein intensity and localisation using IHC in breast cancer cell lines, overall all breast cancer cell lines displayed stronger Ago2 staining, compared to MCF-10A (with very weak staining) (Fig. 1b, Additional file 1: Figure S1B). The Luminal cell lines (T47-D and BT-474) displayed predominantly cytoplasmic Ago2 staining, of low-moderate intensity. The SK-BR3 (HER2 positive) and BT-20 (Triple Negative) cell lines displayed stronger cytoplasmic Ago2 staining (Fig. 1b). Intriguingly, the BT-474, SK-BR-3 and BT-20 lines displayed a sub-population with strong nuclear staining Fig. 1b). Ago2 localisation was explored further using the more sensitive immunofluorescent staining method. In non-dividing cells Ago2 was observed to stain almost exclusively in the cytoplasm in the control MCF10A cells. In non-dividing breast cancer lines there was strong cytoplasmic staining, with additional nuclear staining observed (Additional file 1: Figure S2). Surprisingly, we observed Ago2 localizing to the midbody of dividing cells in MCF10A, T47D, BT-474 and SK-BR-3 (but not in the basal line BT-20: images shown representative of 20 observed mitotics for each cell line), a localisation not previously observed before (Fig. 1c).

### Quantifying Ago2 intensity and localisation in breast cancer samples

**Fig. 2:**
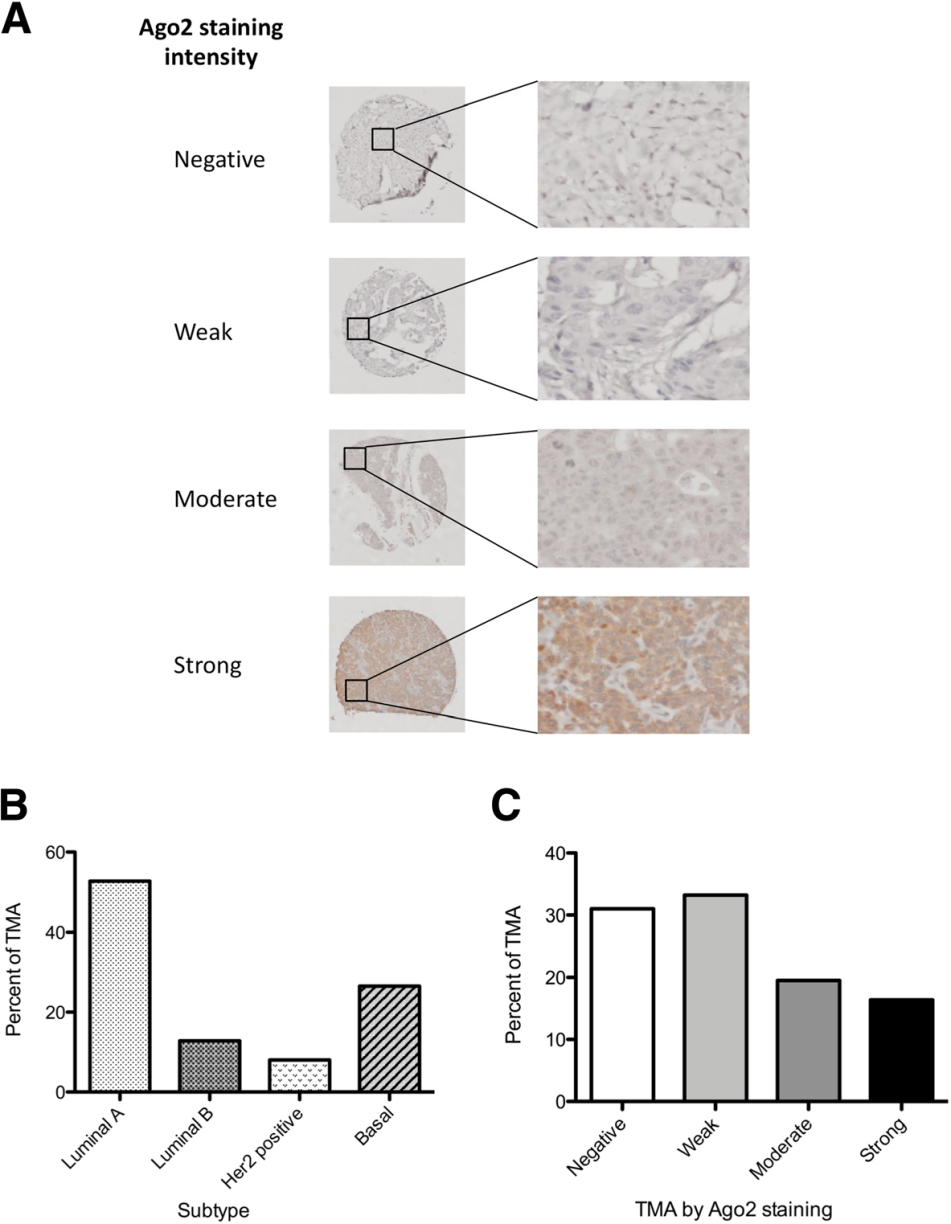
Ago2 staining in breast cancer TMA. **a** Representative staining patters of Ago2 intensity (Negative, Weak, Moderate, Strong). **b** TMA by breast cancer subtype (percent). **c** TMA by Ago2 staining pattern (percent). **b-c** *n* = 226

To further characterize Ago2 localisation in breast cancer, a tissue microarray (TMA) consisting of 328 breast cancer biopsies (Table 1) was stained for Ago2. The intensity of the Ago2 staining observed was categorised (Negative, Weak, Moderate, Strong) (Fig. 2a, Additional file 1: Figure S3). Stratifying the Ago2 stained sections by subtype, we find that the TMA loosely follows the distribution of subtypes observed clinically: Luminal A (52.7%), Luminal B (12.8%), Her2 positive (8%), with a slight overrepresentation of the Basal/Triple Negative (26.5%) subtype (Fig. 2b). Investigating the samples with both subtype and Ago2 staining (*n* = 226), the predominant staining intensity observed was Weak (33.2%, *n* = 75), followed closely by Negative (31%, *n* = 70), Moderate (19.5%, *n* = 44) and Strong (16.4%, *n* = 37) (Fig. 2c). As observed in the breast cancer cell lines, the Ago2 staining pattern observed in the TMA was mainly cytoplasmic (of varying intensities), with nuclear staining observed within the strong staining pattern (Additional file 1: Figure S3, Strong).

### Evaluating Ago2 staining intensity and clinicopathological variables

**Fig. 3:**
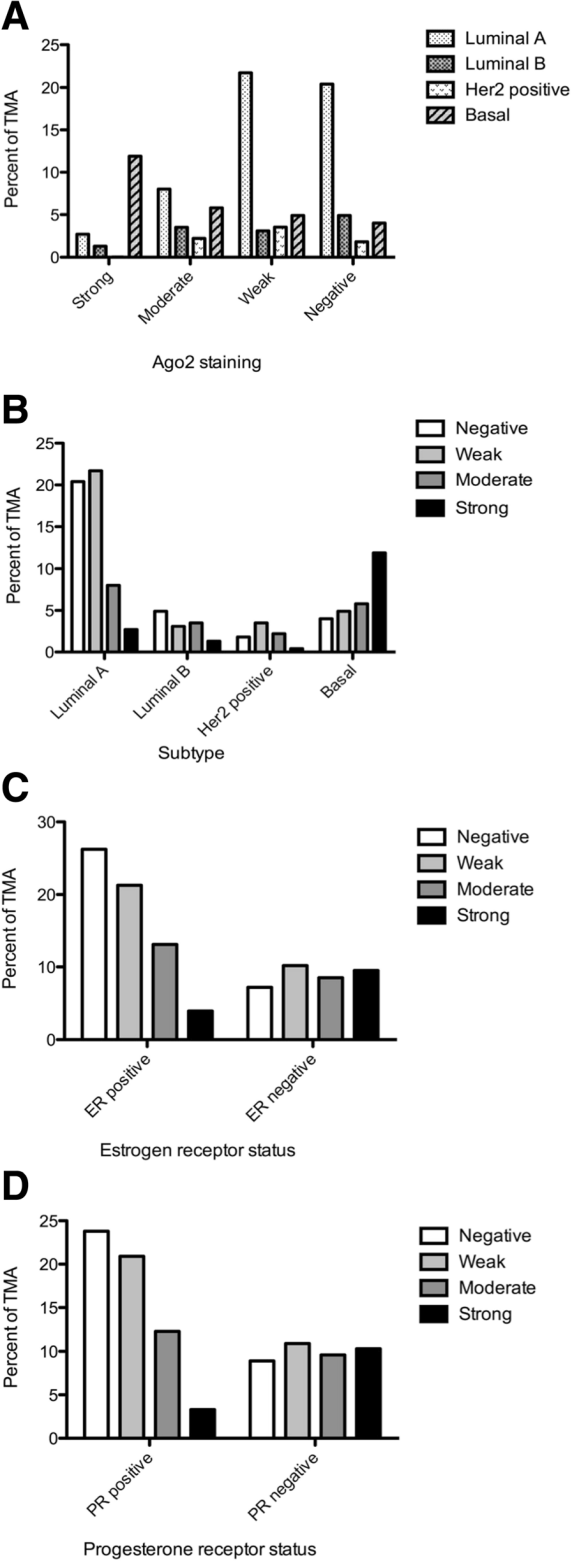
Association of TMA Ago2 intensity and clinicopathological details. **a** TMA by Ago2 staining pattern and breast subtype (percent). **b** TMA by breast subtype and Ago2 staining pattern (percent). **c** Estrogen receptor (ER) positive (percent). Total *n* = 305. ER positive and Ago2 pattern: Negative (*n* = 80), Weak (*n* = 65), Moderate (*n* = 40), Strong (*n* = 12). **d** Progesterone receptor (PR) positive (percent). Total *n* = 302. PR positive and Ago2 pattern: Negative (*n* = 72), Weak (*n* = 63), Moderate (*n* = 37), Strong (*n* = 10)

To explore the relevance of Ago2 staining as a prognostic or diagnostic marker, associations between Ago2 intensity and key clinicopathological factors were tested. Testing the association between Ago2 staining intensity and breast cancer subtype, there is evidence to suggest a general association in the population (p ≈ 0.000) (Fig. 3a-b). Importantly, Goodman and Kruskal’s Lambda (λ) measure for association (a measure of association between two nominal categorical variables) suggests knowledge of Ago2 staining intensity improves the ability to predict breast cancer subtype by 20% (probability distribution analysis of observed and expected counts, determining key variables contributing more to subtype prediction). Testing the association between Ago2 staining intensity and breast cancer receptor status, there is evidence to suggest a general association between the Estrogen Receptor (ER) and Ago2 staining in the population (p ≈ 0.000). Furthermore using the λ measure for association knowledge of Ago2 staining intensity improves the ability to predict ER status by 15.7% (Fig. 3c). There is evidence to suggest a general association between the Progesterone Receptor (PR) and Ago2 staining in the population (p ≈ 0.000). In addition, the λ measure for association suggests knowledge of Ago2 staining improves the ability to predict PR status by 17.5% (Fig. 2d). Interestingly, a higher proportion of ER positive samples with weak or negative staining were observed [combined 73.6% (65 + 80)/197] (Fig. 3c), with a similar trend seen in PR positive samples [combined 74.2% (63 + 72)/182] (Fig. 3d). Intriguingly, if we consider only the ER or PR negative groups (Fig. 3c-d), the average of the Ago2 staining intensities would be relatively similar, averaging 8.85% (±1.3%) and 9.93% (±0.87%) respectively, suggesting a possible link between ER/PR positivity and Ago2 regulation.

**Fig. 4:**
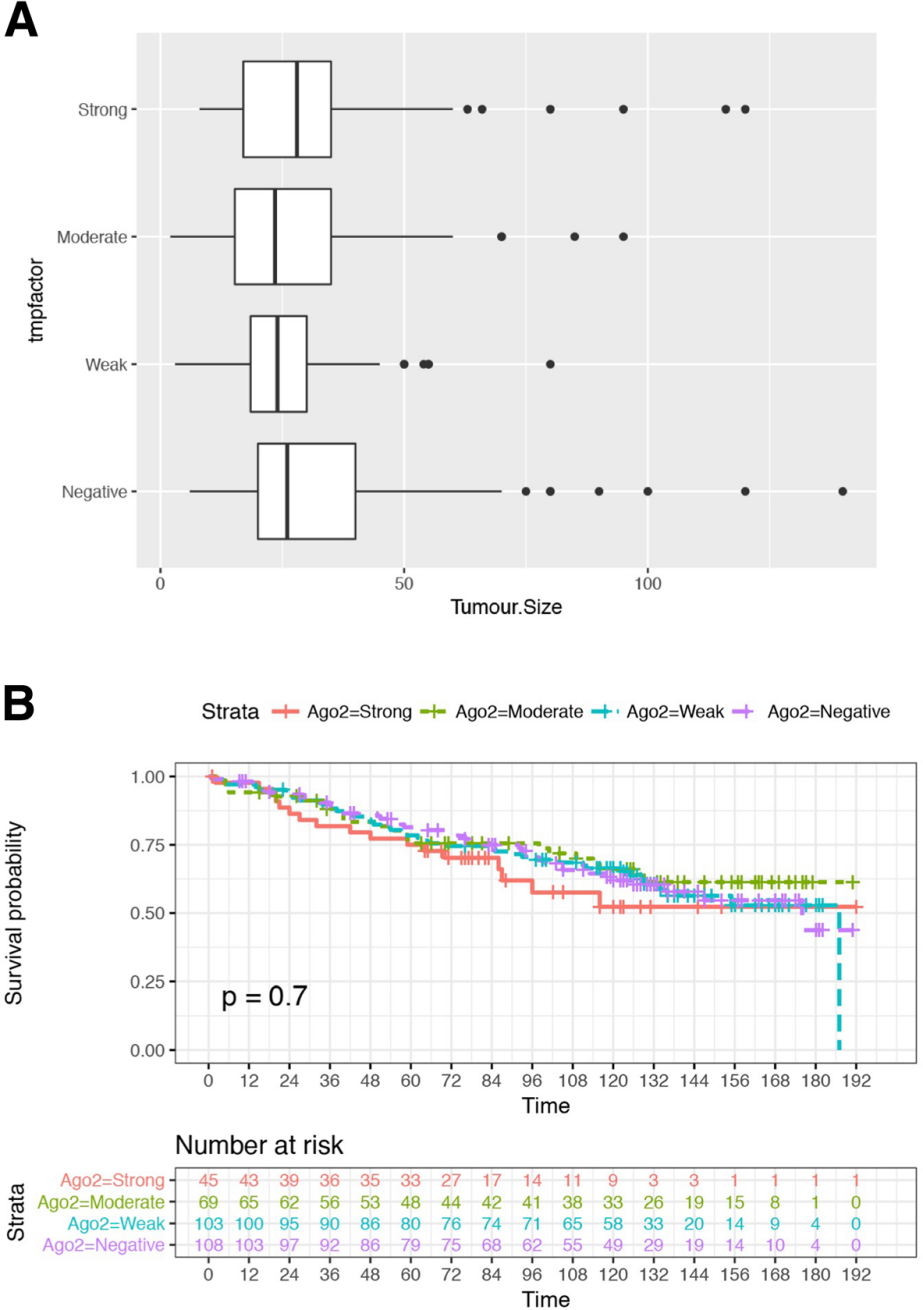
Association of TMA Ago2 intensity and further clinicopathological details. **a** Association of TMA Ago2 intensity and Tumour size (mm). Boxplots by Ago2 staining intensity. Total *n* = 312. Negative (*n* = 106), Weak (*n* = 99), Moderate (*n* = 62), Strong (*n* = 45). **b** Association of TMA Ago2 intensity and survival. *N* = 325. *p* = 0.7

Exploring the relationship between Ago2 staining intensity and clinicopathological variables, the Kruskal-Wallis test (a rank-based nonparametric test used to determine any statistically significant differences between two or more populations) suggests a significant difference in the tumour size distribution, when comparing Ago2 staining intensities (*p* = 0.0267) (Fig. 4a). The observed data gave no evidence of an association between Ago2 staining intensity with the other commonly tested clinicopathological variables (T-score, N-score, M-score, NPI, DCIS, Her2, Stage or age) (Data not shown). There is no evidence of an effect of Ago2 staining intensity on Overall Survival (OS) outcome, comparing the different Ago2 staining patterns in the population (*p* = 0.7) (Fig. 4b). Additionally, there is no evidence of an effect of Ago2 staining intensity on Disease-Free Survival (DFS) (Data not shown).

### Modeling Ago2 staining intensities and survival

To further analyze the relationship between Ago2 staining intensity and the common clinicopathological variables two types of saturated models (with are as many estimated parameters as data points) were utilized: Complete-case and Imputed-dataset. Complete-case uses and analyzes only data from patients with complete covariate data set, which can be considered inefficient as not all data in the cohort is utilized. However, the Imputed-dataset modeling can incorporate data from all cohort patients (featuring a predictive model based on observed data to estimate missing covariate values). Investigating the fitting of complete-case saturated and Imputed-dataset saturated models for DFS, the models fitted 177 (of 308) complete-case individuals across the modelled variables [Ago2 staining intensity, Subtype, Nottingham Prognostic Index (NPI), Tumour size, disease stage, patient age] (Additional file 1: Figure S4A and B). As expected in this model Basal Subtype and Stage III show a significant difference in DFS outcome (*p* = 0.02 and 0.02 respectively). In these models comparing different Ago2 staining patterns in the population found no significance.

### Investigating Ago2 mRNA expression and DFS or OS in breast cancer

**Fig. 5:**
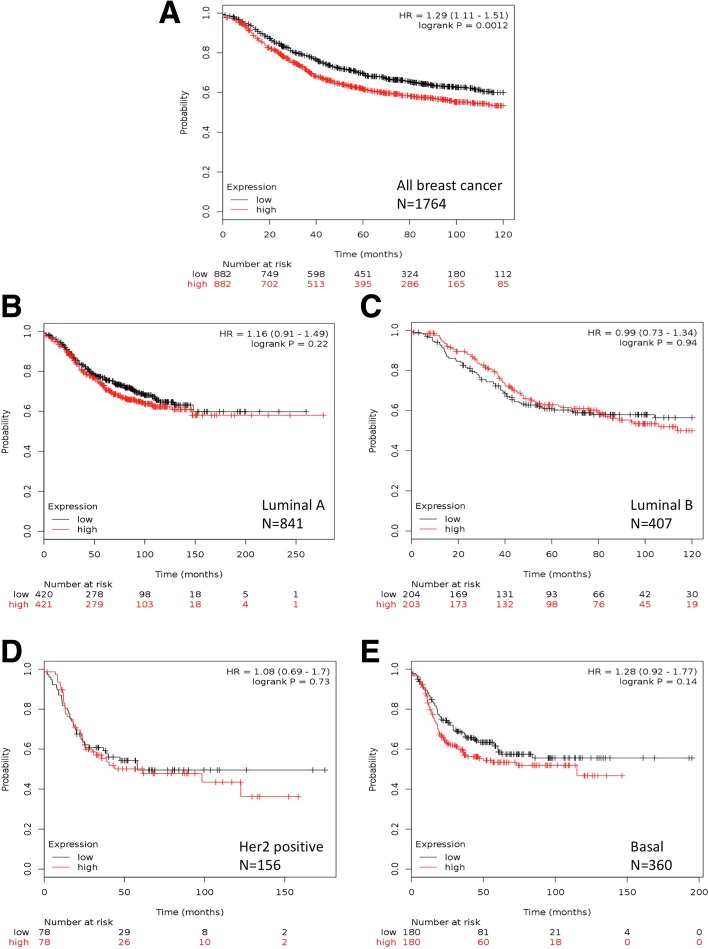
Ago2 mRNA expression and Disease Free Survival. **a** All breast cancer subtypes (*n* = 1764). **b** Luminal A Breast cancers (*n* = 841). **c** Luminal B Breast cancers (*n* = 407). **d** Her2 positive Breast cancers (*n* = 156). **e** Basal Breast cancers (*n* = 360). Using only JetSet best probe set. Generated using Kaplan-Meier plotter [16]

It was previously reported that Ago2 protein and mRNA levels were differentially regulated in melanoma [32]. Therefore, the expression levels of Ago2 mRNA in breast cancer were investigated [16, 23, 28–32]. Ago2 mRNA expression levels were correlated this with key clinicopathological characteristics DFS and OS [16, 33, 34]. Investigating Ago2 mRNA expression and DFS, we find evidence of a difference in DFS comparing Ago2 expression Low and High, when considering all breast cancers (*p* = 0.0012, *n* = 1764) (Fig. 5a). Investigating individual subtypes, no evidence of a difference in DFS comparing Ago2 expression Low and High was found [Luminal A, *p* = 0.22; Luminal B, *p* = 0.94; Her2 positive, *p* = 0.73; Basal, *p* = 0.14] (Fig. 5b-e). Comparing the distribution of OS between High and Low Ago2 expression, considering all breast cancers (including all breast cancer subtypes, *n* = 626) there is no evidence of a difference (*p* = 0.5608) (Additional file 1: Figuere S5A). Furthermore, there was no evidence of a difference in OS between High and Low Ago2 expression within each individual subtype [Luminal A, *p* = 0.5941; Luminal B, *p* = 0.8123; Her2 positive, *p* = 0.2194; Basal, *p* = 0.4965] (Additional file 1: Figure S5B-E).

Previous work found that increased Ago2 mRNA expression was associated with increased AGO2 gene copy number, and this was significantly associated with a poorer disease outcome in high-risk multiple myeloma [36]. Using the cBioPortal [20, 29] we investigated copy number variations (CNVs) of the Ago2 gene in breast cancer. Amplification of the Ago2 gene was found in 18.25% of cases (650/3562 samples), in data from seven independent breast databases available (with more than 100 samples), ranging between ~ 27–10% (in individual studies, where amplification was observed) (Additional file 1: Figure S6A). Importantly, no significant Ago2 gene deletion was observed in this breast cancer cohort (4/3562 samples, 0.11%). Interestingly, a very low Somatic Mutation Frequency (missense) of 0.31% (*n* = 11) was observed (Additional file 1: Figure S6B).

## Discussion

1. Currently, breast cancer is stratified into four main subtypes, however up to ten molecular subtypes have been described in the literature [37, 38]. The continual advancement of our molecular understanding of breast cancer, through the profiling of new markers, is allowing us to better stratify and predict the outcome of breast cancer. To achieve this, further molecular characterisation of breast cancer is required to allow new markers, such as proteins like Ago2, to improve or enhance current stratifications and reveal further underlying molecular changes influencing breast cancer cells.

It has been previously recognized that quantifying levels of the miRNA processing machinery has the ability to act as diagnostic or prognostic markers in some cancer types [23]. Quantifying the miRNA machinery, and exploring its effects on breast cancer, holds particular relevance for evaluating any potential future use of miRNA as a therapeutic strategy. Here we investigated the value of evaluating Ago2 levels (protein and transcript) as markers in breast cancer. Results from representative cell lines indicate that while total levels of Ago2 protein do not vary as much as transcript levels, there were significant differences in the intensity and localisation of Ago2 protein histological staining. The apparent disconnect between transcript and protein levels suggests that the turnover/stability of Ago2 protein is tightly and differentially regulated in breast cancer subtypes, as has previously been demonstrated in ER positive cells, which can in turn influence growth rates [29]. Further work is needed to characterise the molecular mechanisms that regulate Ago2 localisation and stability in breast cancer cells. The staining in the representative cell lines correlated with the protein patterns seen in the TMA, suggesting that the cell lines are a good model to further explore the role of Ago2 in breast cancer. Of great interest in the cell lines was our surprising result showing for the first time Ago2 localizing to the midbody. This result, combined with the sub-population displaying nuclear staining (by IHC and IF), suggests that Ago2 expression (at least in these lines) may be cell cycle regulated. Importantly, a cell cycle regulated role (possibly miRNA independent) for Ago2 in cell division could have implications for further understanding mechanisms supporting mitotic defects and aneuploidy (if Ago2 is essential for cell division). Indeed, this is supported by previous work that found silencing of Ago2 resulted in a G2/M arrest in prostate cancer cells [31]. Further work to investigate this potential new role could reveal additional core roles of Ago2 that impact on tumour progression.

We found that knowing Ago2 protein levels in tumour improves the ability to predict breast cancer subtype (by 20%), which is related Ago2 staining improving the ability to predict Estrogen or Progesterone receptor status (by 15.7 and 17.5% respectively). Overall, using a larger cohort (through the online repositories) we found that high Ago2 mRNA expression correlates with a poor relapse free survival in breast cancer. This supports previous work that reported that high Ago2 protein expression in Glioma and correlates with poorer survival [30], suggesting potentially that further work may establish a common role for Ago2 in cancer progression. A miRNA dependent role for Ago2 has been established in tumorigenesis and its miRNA-independent roles have been linked to cancer initiation/progression through interactions with known tumour promoting factors (FAK, SWI/SNF, KRAS) [4, 39–43]. Together these point to a role for Ago2 (either directly or indirectly) in promoting or regulating tumour growth.

It has previously been demonstrated that Ago2 expression (mRNA and protein) can be regulated by the Estrogen receptor mediated pathway in cancer cell lines [29], and it was interesting to note that we saw lower and more variable levels of Ago2 protein expression seen in the ER positive cancer samples (with unchanging Ago2 levels in ER or PR negative patient samples). We investigated the expression of Ago2 mRNA and protein in breast cancer and found that there were distinct patterns of Ago2 protein expression, however a larger cohort will be needed to confirm if these patterns are significant. Combined with Ago2 mRNA expression data demonstrating a correlation with DFS overall, we believe that measuring both Ago2 mRNA and protein levels may hold further promise in aiding the stratification of breast cancer.

Previous work demonstrated that changes in Ago2 gene copy can affect expression levels and correlate with high-risk disease in multiple myeloma [36]. In breast cancer we found high levels of Ago2 gene amplification (almost 18%) and that high Ago2 expression significantly correlated with an increased chance of relapse. Future work should explore Ago2 expression levels in the tumour samples with gene amplification. Combining the previous findings and our results it is possible that investigation of the specific cohort of patients with gene amplification may reveal if this cohort is indeed a high-risk group. Further molecular work investigating the cellular consequences, of both up and down regulation of Ago2 are needed to clarify its role in breast cancer. In breast cancer, single-nucleotide polymorphisms of Ago2 have been associated with changes in disease free survival (DFS) and overall survival (OS) [33]. We found a limited number of SNPs in our searches, however further work will be required to determine what effects these specific SNPs have on the regulation or function of Ago2, as some SNPs have previously been shown to exert effects in ovarian cancer [44].

## Conclusions

We demonstrate that quantification of Ago2 protein levels in breast tumour samples correlates with tumour size, ER and PR status and that this can be used to improve the ability to predict breast cancer subtype (by 20%). Therefore, Ago2 quantification could be used to improve subtype classification in heterogeneous tumours, or tumours with borderline ER/PR staining. Reduced expression of Ago2 is associated with poor patient survival, and Ago2 may be useful as part of future prognostic gene panels or stratification signatures. Together with the previously published data, our work supports further investigation of the role of Ago2, both miRNA dependent and independent, as a relevant factor influencing breast cancer (initiation, maintenance or progression). The role of Ago2 and ER/PR expression requires further investigation, to determine if this is a cause or consequence of the poor clinical outcome observed, and if this is related to any chemotherapy-mediated resistance mechanisms [44]. This supports further investigation of Ago2 quantification as a method for identifying patients likely to have a poorer clinical outcome, which would improve prognostic/diagnostic evaluations.

## Additional file

Additional file 1: **Figure S1.** Ago2 expression in breast cancer cell lines. **A.** Ago2 mRNA expression in breast cancer cell lines. *N* = 3 independent experiments. Endogenous control genes used: Mitochondrial Ribosomal Protein L19 (MRPL19) and Peptidylprolyl Isomerase A (PPIA). A *P*-value < 0.05 (*) was deemed significant. **Figure S2**. Immunofluorescence staining of Ago2 in breast cell lines. **A.** Indicated breast cell lines were fixed and stained for Ago2 (red) and DNA counterstained with DAPI (blue). Scale bar, 20um. **Figure S3. A.** Negative control Ago2 IHC staining. **B.** Multiple representative images of Ago2 staining pattern in TMA. **Figure S4. A.** Fitting of complete-case saturated model for Disease Free Survival. **B.** Fitting of imputed-dataset saturated model for Disease-Free Survival. **Figure S5.** Ago2 mRNA expression and Overall Survival. **A.** All breast cancer subtypes (*n* = 626). **B.** Luminal A Breast cancers (*n* = 271). **C.** Luminal B Breast cancers (*n* = 129. **D.** Her2 positive Breast cancers (*n* = 73). **E.** Basal Breast cancers (*n* = 153). Using only JetSet best probe set. Censure at threshold 10 years. Generated using Kaplan-Meier plotter [16]. **Figure S6.** Genomic changes observed Ago2 gene in breast cancer. **A.** Ago2 gene amplification in indicated Breast cancer databases. **B.** Ago2 mutations observed in breast cancer. (PDF 4035 kb)

## Data Availability

The datasets generated for this study are available from the corresponding author on reasonable request.

## Abbreviations

Ago2: Argonaute 2
DFS: Disease free survival
EIF2C2: Eukaryotic translation initiation factor 2C
ER: Estrogen receptor
IHC: Immunohistochemical staining
miRNA: MicroRNA
mRNA: Messenger RNA
PR: Progesterone receptor
RFS: Relapse free survival
