# Supplementary material for: Quantifying Argonaute 2 (Ago2) expression to stratify breast cancer
Source: BMC Cancer. 2019 Jul 19;19:712. doi: 10.1186/s12885-019-5884-x (PMC6642579; doi:10.1186/s12885-019-5884-x)
Supplement: Supplementary file 1 — Figure S1. Ago2 expression in breast cancer cell lines. A. Ago2 mRNA expression in breast cancer cell lines. N = 3 independent experiments. Endogenous control genes used: Mitochondrial Ribosomal Protein L19 (MRPL19) and Peptidylprolyl Isomerase A (PPIA). A P-value < 0.05 (*) was deemed significant. Figure S2. Immunofluorescence staining of Ago2 in breast cell lines. A. Indicated breast cell lines were fixed and stained for Ago2 (red) and DNA counterstained with DAPI (blue). Scale bar, 20um. Figure S3. A. Negative control Ago2 IHC staining. B. Multiple representative images of Ago2 staining pattern in TMA. Figure S4. A. Fitting of complete-case saturated model for Disease Free Survival. B. Fitting of imputed-dataset saturated model for Disease-Free Survival. Figure S5. Ago2 mRNA expression and Overall Survival. A. All breast cancer subtypes (n = 626). B. Luminal A Breast cancers (n = 271). C. Luminal B Breast cancers (n = 129. D. Her2 positive Breast cancers (n = 73). E. Basal Breast cancers (n = 153). Using only JetSet best probe set. Censure at threshold 10 years. Generated using Kaplan-Meier plotter [16]. Figure S6. Genomic changes observed Ago2 gene in breast cancer. A. Ago2 gene amplification in indicated Breast cancer databases. B. Ago2 mutations observed in breast cancer. (PDF 4035 kb) [file 12885_2019_5884_MOESM1_ESM.pdf]

A

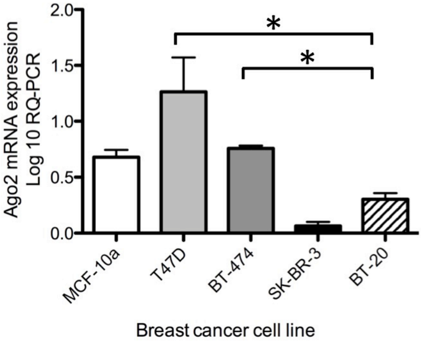

B

Negative Control
(secondary antibody alone)

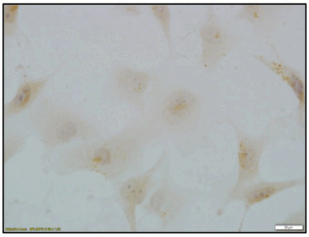

Figure S1

A

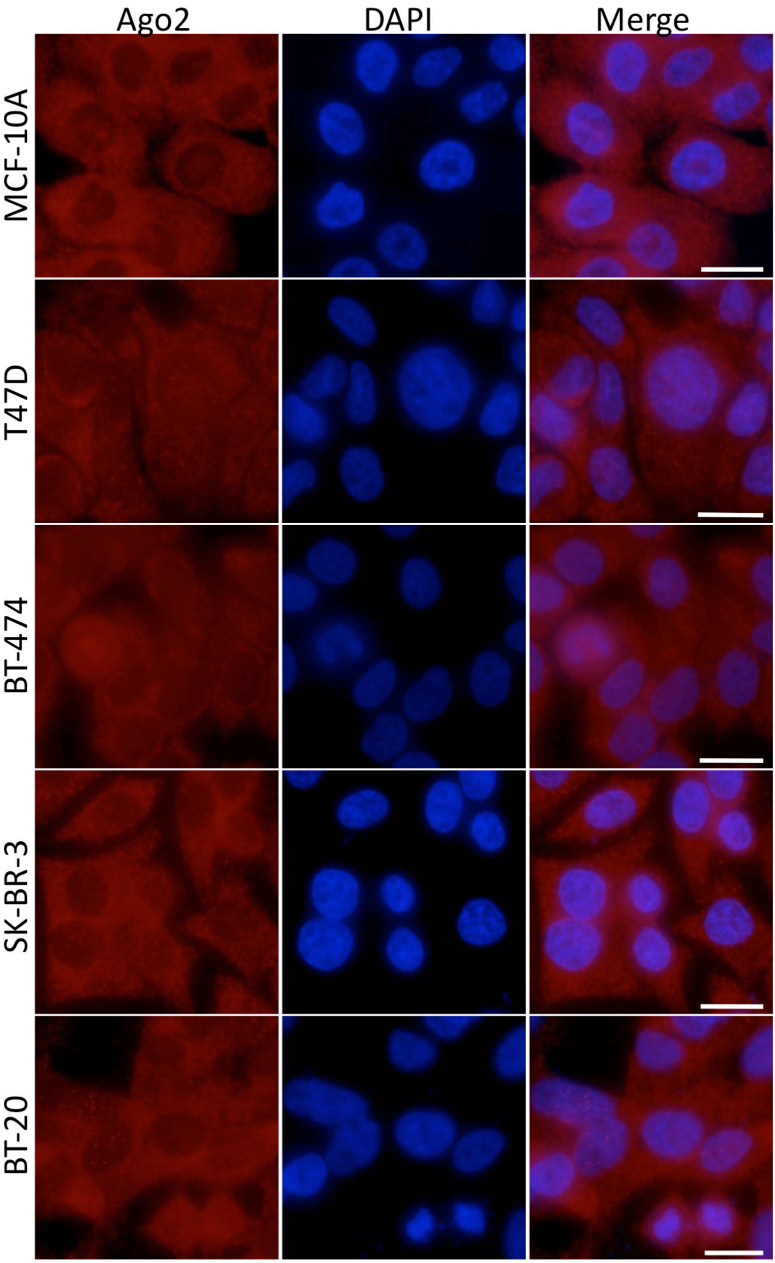

Figure S2

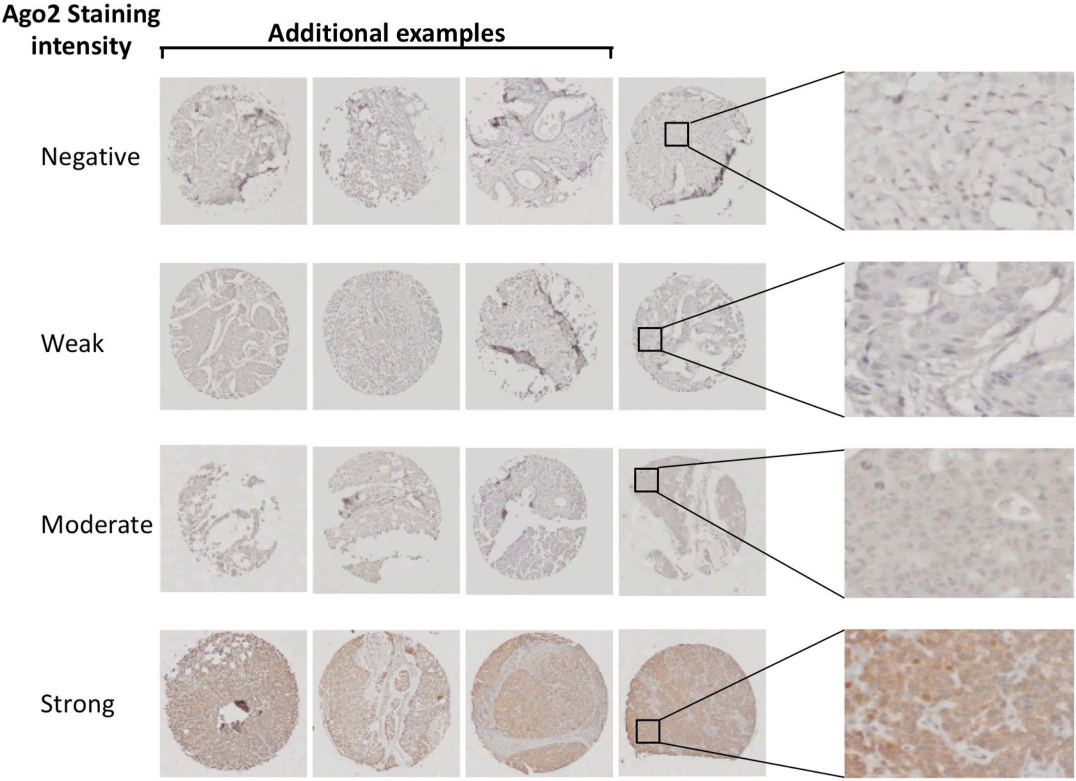
Ago2 Staining intensity
Additional examples
Negative
Weak
Moderate
Strong

Figure S3

A

Fitting of complete-case saturated model for Disease Free Survival

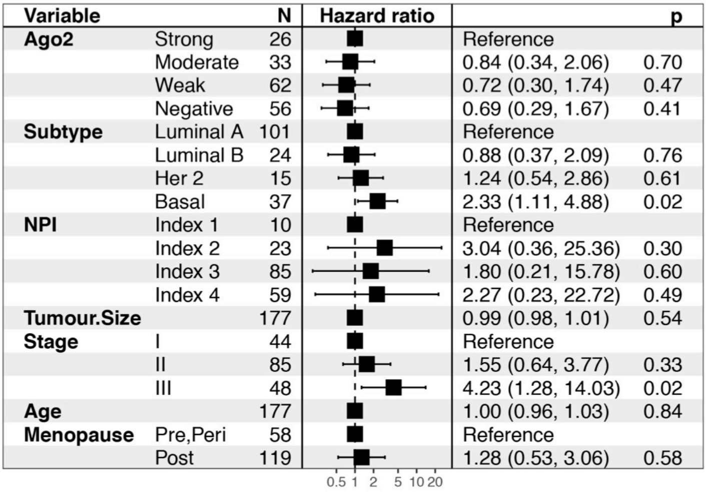

Variable		N	Hazard ratio		p
**Ago2**	Strong	26		Reference	
	Moderate	33		0.84 (0.34, 2.06)	0.70
	Weak	62		0.72 (0.30, 1.74)	0.47
	Negative	56		0.69 (0.29, 1.67)	0.41
**Subtype**	Luminal A	101		Reference	
	Luminal B	24		0.88 (0.37, 2.09)	0.76
	Her 2	15		1.24 (0.54, 2.86)	0.61
	Basal	37		2.33 (1.11, 4.88)	0.02
**NPI**	Index 1	10		Reference	
	Index 2	23		3.04 (0.36, 25.36)	0.30
	Index 3	85		1.80 (0.21, 15.78)	0.60
	Index 4	59		2.27 (0.23, 22.72)	0.49
**Tumour.Size**		177		0.99 (0.98, 1.01)	0.54
**Stage**	I	44		Reference	
	II	85		1.55 (0.64, 3.77)	0.33
	III	48		4.23 (1.28, 14.03)	0.02
**Age**		177		1.00 (0.96, 1.03)	0.84
**Menopause**	Pre,Peri	58		Reference	
	Post	119		1.28 (0.53, 3.06)	0.58

B

Fitting of imputed-dataset saturated model for Disease-Free Survival

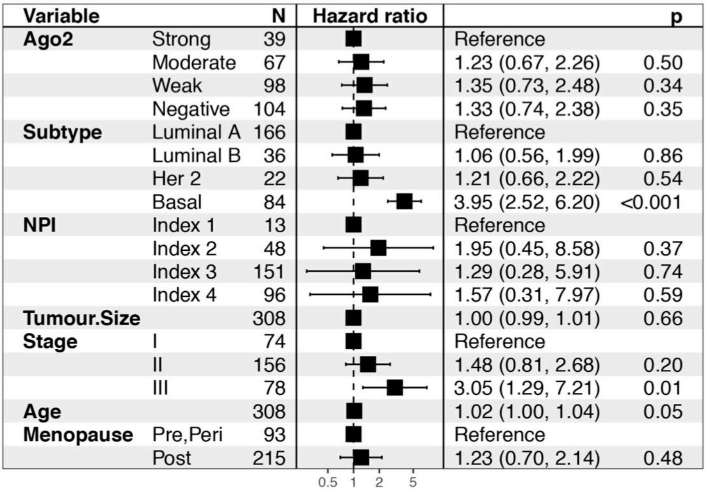

Variable		N	Hazard ratio		p
**Ago2**	Strong	39		Reference	
	Moderate	67		1.23 (0.67, 2.26)	0.50
	Weak	98		1.35 (0.73, 2.48)	0.34
	Negative	104		1.33 (0.74, 2.38)	0.35
**Subtype**	Luminal A	166		Reference	
	Luminal B	36		1.06 (0.56, 1.99)	0.86
	Her 2	22		1.21 (0.66, 2.22)	0.54
	Basal	84		3.95 (2.52, 6.20)	<0.001
**NPI**	Index 1	13		Reference	
	Index 2	48		1.95 (0.45, 8.58)	0.37
	Index 3	151		1.29 (0.28, 5.91)	0.74
	Index 4	96		1.57 (0.31, 7.97)	0.59
**Tumour.Size**		308		1.00 (0.99, 1.01)	0.66
**Stage**	I	74		Reference	
	II	156		1.48 (0.81, 2.68)	0.20
	III	78		3.05 (1.29, 7.21)	0.01
**Age**		308		1.02 (1.00, 1.04)	0.05
**Menopause**	Pre,Peri	93		Reference	
	Post	215		1.23 (0.70, 2.14)	0.48

Figure S4

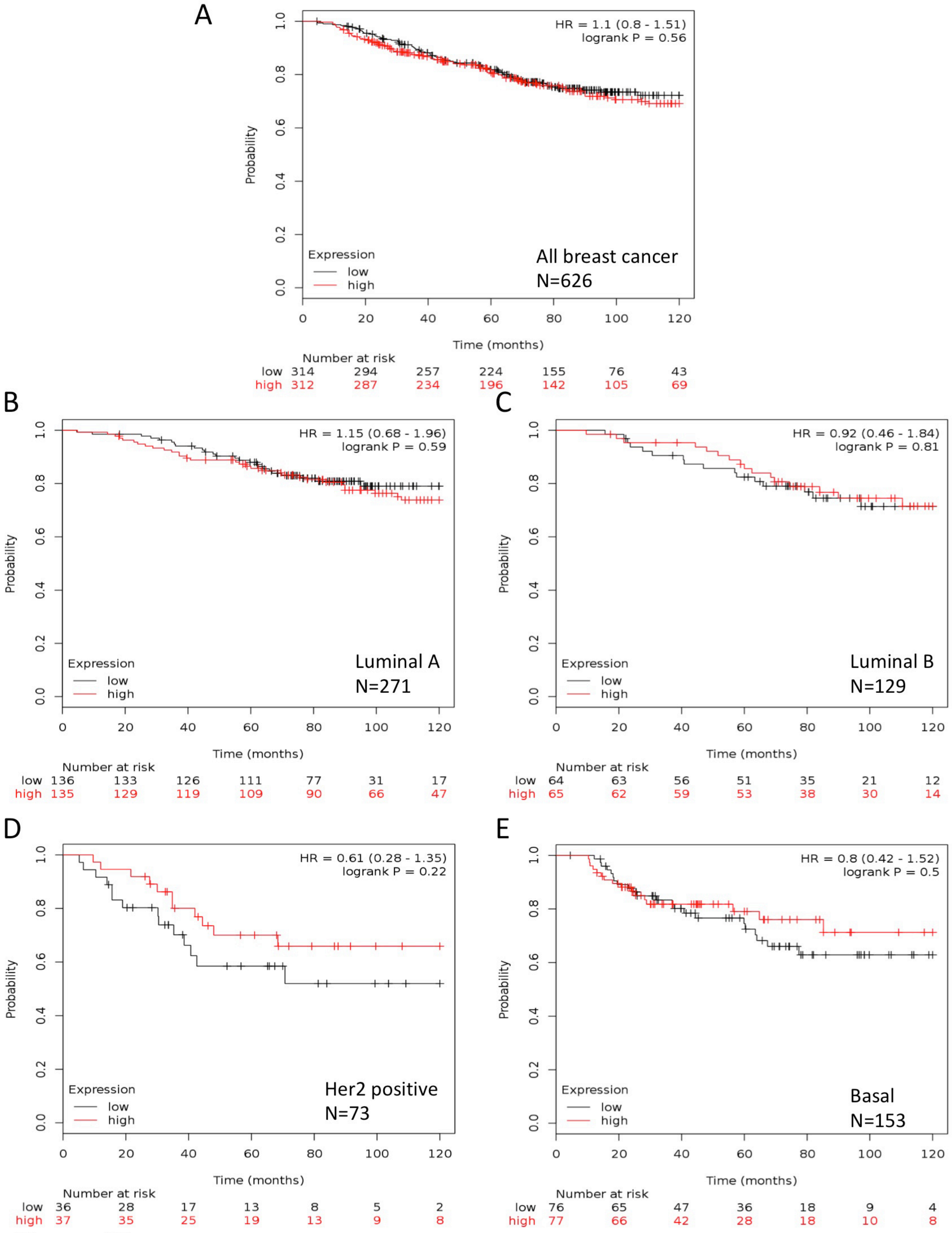
A
HR = 1.1 (0.8 - 1.51)
logrank P = 0.56
Probability
Expression
low
high
All breast cancer
N=626
Time (months)
Number at risk
low 314 294 257 224 155 76 43
high 312 287 234 196 142 105 69
B
HR = 1.15 (0.68 - 1.96)
logrank P = 0.59
Luminal A
N=271
Number at risk
low 136 133 126 111 77 31 17
high 135 129 119 109 90 66 47
C
HR = 0.92 (0.46 - 1.84)
logrank P = 0.81
Luminal B
N=129
Number at risk
low 64 63 56 51 35 21 12
high 65 62 59 53 38 30 14
D
HR = 0.61 (0.28 - 1.35)
logrank P = 0.22
Her2 positive
N=73
Number at risk
low 36 28 17 13 8 5 2
high 37 35 25 19 13 9 8
E
HR = 0.8 (0.42 - 1.52)
logrank P = 0.5
Basal
N=153
Number at risk
low 76 65 47 36 18 9 4
high 77 66 42 28 18 10 8

Figure S5

A

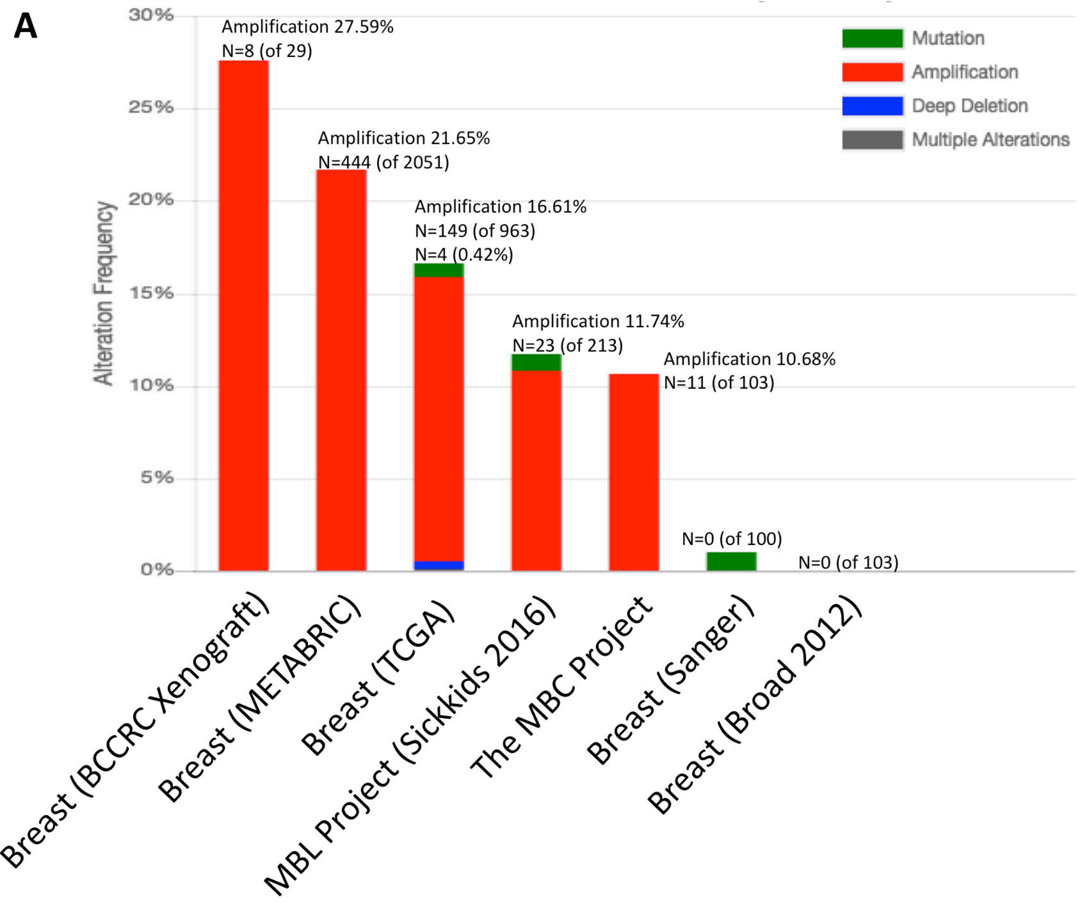

B

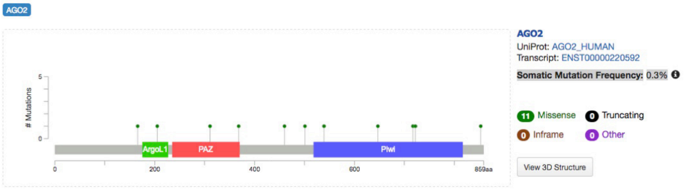

11 Mutations (page 1 of 1)

Study	Sample ID	Cancer Type	Protein Change	Annotation ▼	Mutation Type	Copy #	COSMIC	Allele Freq (T)	# Mut in Sample
Breast Invasive ...	TCGA-EW-A3E8-01	Breast Invasive Lobular Carcin...	R647H		Missense	Diploid			67
Breast Invasive ...	TCGA-A2-A0CV-01	Breast Invasive Ductal Carcinoma	R460C		Missense	Gain			19
Breast Invasive ...	TCGA-A2-A0CW-01	Breast Invasive Ductal Carcinoma	T368S		Missense	Amp			50
Breast Invasive ...	TCGA-A2-A0SY-01	Breast Invasive Lobular Carcin...	S205F		Missense	Diploid			98
Breast Invasive ...	TCGA-AQ-A04H-01	Breast Invasive Ductal Carcinoma	C717S		Missense	Gain			51
Breast Invasive ...	TCGA-E2-A15M-01	Breast Invasive Lobular Carcin...	E722V		Missense	Gain			49
Breast Invasive ...	TCGA-A2-A0T5-01	Breast Invasive Ductal Carcinoma	V539G		Missense	Gain			1094
Breast Invasive ...	TCGA-D8-A1XK-01	Breast Invasive Ductal Carcinoma	Y311H		Missense	Gain			727
Breast Invasive ...	PD4203a	Invasive Breast Carcinoma	L853V		Missense				466
Mutational profi...	MBC_48	Invasive Breast Carcinoma	V501M		Missense	Diploid		0.50	83
Mutational profi...	MBC_188	Invasive Breast Carcinoma	M166I		Missense	Diploid		0.31	248

Showing 1-11 of 11 Mutations

Figure S6
